# Maize Leaf Disease Recognition Based on Improved Convolutional Neural Network ShuffleNetV2

**DOI:** 10.3390/plants13121621

**Published:** 2024-06-12

**Authors:** Hanmi Zhou, Yumin Su, Jiageng Chen, Jichen Li, Linshuang Ma, Xingyi Liu, Sibo Lu, Qi Wu

**Affiliations:** 1 College of Agricultural Equipment Engineering, Henan University of Science and Technology, Luoyang 471003, China; symm1037@163.com (Y.S.); cjg980227171x@163.com (J.C.); lijichenli@163.com (J.L.); mls15036005926@163.com (L.M.); liuxingyi5742@163.com (X.L.); 20143300216@stu.haust.edu.cn (S.L.); 2 College of Water Conservancy, Shenyang Agricultural University, Shenyang 110866, China

**Keywords:** precision agriculture, deep learning, plant diseases, convolutional neural network

## Abstract

The occurrence of maize diseases is frequent but challenging to manage. Traditional identification methods have low accuracy and complex model structures with numerous parameters, making them difficult to implement on mobile devices. To address these challenges, this paper proposes a corn leaf disease recognition model SNMPF based on convolutional neural network ShuffleNetV2. In the down-sampling module of the ShuffleNet model, the max pooling layer replaces the deep convolutional layer to perform down-sampling. This improvement helps to extract key features from images, reduce the overfitting of the model, and improve the model’s generalization ability. In addition, to enhance the model’s ability to express features in complex backgrounds, the Sim AM attention mechanism was introduced. This mechanism enables the model to adaptively adjust focus and pay more attention to local discriminative features. The results on a maize disease image dataset demonstrate that the SNMPF model achieves a recognition accuracy of 98.40%, representing a 4.1 percentage point improvement over the original model, while its size is only 1.56 MB. Compared with existing convolutional neural network models such as EfficientNet, MobileViT, EfficientNetV2, RegNet, and DenseNet, this model offers higher accuracy and a more compact size. As a result, it can automatically detect and classify maize leaf diseases under natural field conditions, boasting high-precision recognition capabilities. Its accurate identification results provide scientific guidance for preventing corn leaf disease and promote the development of precision agriculture.

## 1. Introduction

Maize (*Zea mays* L.), commonly referred to as corn, stands as a fundamental crop with a rich history of sustaining human civilizations over millennia [1]. Originating from Mesoamerica, maize has evolved into a global staple, indispensable not only for human consumption but also for animal feed, biofuel production, and various industrial applications. According to the Food and Agriculture Organization Statistics (FAOSTAT), maize ranks among the most cultivated cereals globally, with a staggering production of approximately 1.14 billion tons in 2019 [2]. In the United States, maize holds the title of the most produced crop, occupying over 92 million acres of cultivated land [3]. This widespread cultivation underscores maize’s pivotal role in global food security and agricultural economies. However, maize is frequently threatened by a range of pests and diseases, including maize leaf spot disease, pathogenic spot disease, maize aphids, and maize borers [4]. These challenges not only compromise yield quantity and quality but also jeopardize food security. To ensure successful maize cultivation, comprehensive measures must be taken to manage pests and diseases.

At present, traditional methods of pest and disease identification rely on manual observation and empirical judgment, processes that are time-consuming and prone to human error [5]. Professionals are tasked with identifying and categorizing maize leaves through the visual inspection or microscopic examination of features such as morphology, color, and texture [6]. Complex backgrounds pose a significant challenge in these traditional methods due to visual noise, similarity in color and texture between disease symptoms and background elements, and dynamic environmental changes that can obscure or alter the appearance of disease symptoms. Advancements in computer vision and image processing technologies have revolutionized crop disease identification, offering more efficient and objective avenues for disease detection through automated methods leveraging techniques like feature extraction, image segmentation, and machine learning [7,8]. The identification of pests and diseases using leaf images has emerged as a critical domain in plant health detection [9].

In the 21st century, convolutional neural networks (CNNs), a deep learning technique, have achieved remarkable success in image-based recognition tasks [10]. A CNN autonomously extracts image features and classifies images based on these features [11]. For maize leaf disease recognition, researchers commonly employ CNNs to train on maize leaf images, discerning maize leaf disease types by learning image features [12]. Specifically, CNN scrutinizes maize leaf image intricacies and characteristics, mapping them to maize leaf disease categories, thereby achieving rapid and precise disease recognition and classification. However, the presence of complex backgrounds in leaf images further complicates this task. Background elements such as soil, other plants, and varying lighting conditions can introduce noise that confounds the disease detection algorithms. Successful models must therefore effectively differentiate between disease symptoms and irrelevant background details. In recent years, an increasing number of researchers have embraced CNN techniques for crop leaf disease identification, yielding significant outcomes [13]. For instance, Hlaing et al. [14] characterized tomato images using the Johnson SB distribution model, achieving an average accuracy of 85.1%. Junde et al. [15] integrated channel attention into the lightweight neural network model MobileNetV2, enhancing pest recognition in complex backgrounds, with an average accuracy of 92.79%. Yun et al. [16] embedded an improved channel and spatial attention module into ResNet, achieving an average accuracy surpassing 95.37%. Hidayatuloh et al. [17] utilized the Keras deep learning framework to enhance the SqueezeNet model for automatic disease detection in tomato leaf images, boasting an average recognition accuracy of 86.92%. Agarwal et al. [18] proposed a model for tomato leaf disease recognition and detection, achieving an average accuracy of 91.2%. Bhujel et al. [19] devised a lightweight CNN integrating various attention modules to bolster overall accuracy, validated on a tomato leaf disease dataset. Bari et al. [20] employed Faster R-CNN for the real-time detection of rice leaf diseases, achieving accuracies of 98.09%, 98.85%, and 99.17%. Trivedi et al. [21] utilized Google Testbed on a dataset containing tomato leaf samples, attaining a prediction accuracy of 98.49%. Deepalakshmi et al. [22] developed a CNN model capable of recognizing various image types with an average accuracy of 94.5% and a recognition cost of 3.8 s. Sibiya et al. [23] devised a tomato leaf disease detection model based on ResNet50 using PyTorch, achieving 97% accuracy.

Leveraging CNN technology for maize leaf disease identification holds immense significance, substantially reducing diagnosis time and enhancing diagnosis accuracy and efficacy, thereby positively impacting maize yield and quality [24]. Therefore, this paper proposes a CNN model (SNMPF), which is based on the improved ShuffleNetV2 and aims to achieve the high-precision recognition of maize leaf disease. By optimizing the network structure, the SNMPF model can improve the recognition performance of corn leaf disease. At the same time, the lightweight design of the model enables it to be deployed on mobile devices, facilitating its practical application in the field. It provides farmers with a new technological means for the timely detection and prevention of diseases and pests, thereby reducing grain yield losses.

Therefore, the focus of this paper is to achieve high accuracy in maize leaf disease recognition, as well as a lightweight model. To accomplish this, the lightweight convolutional neural network ShuffleNetV2 was utilized, and the following studies and improvements were conducted:

- Augmentation of data using the Augmentor tool, employing random combinations of operations such as random rotation, vertical flip, morphological magnification, region erasure, and brightness transformation to bolster model training generalization ability;
- Division of data into training and validation sets in a 4:1 ratio, with 3200 images allocated for training and 800 sheets for validation;
- Comparative study of four models, ShuffleNetV2, ShuffleNetV2 + Max pooling (MP), ShuffleNetV2 + SimAM (SAM), and ShuffleNetV2 + Max pooling (MP) + SimAM (SAM), to assess the contributions of different improvement measures;
- Comparison between SimAM and the attention mechanisms, including SE, ECA, EMA, and CSAM. The suitability of each attention mechanism for the task of maize leaf disease recognition is being determined through comparison;
- Comparative evaluation with existing network models such as EfficientNet, Reg-Net, MobileViT, EfficientNetV2, and DenseNet showcases the advantages of the SNMPF lightweight model in maize leaf disease identification amidst complex backgrounds.

## 2. Materials and Methods

### 2.1. Data Selection and Preprocessing

#### 2.1.1. Image Data

**Figure 1:**
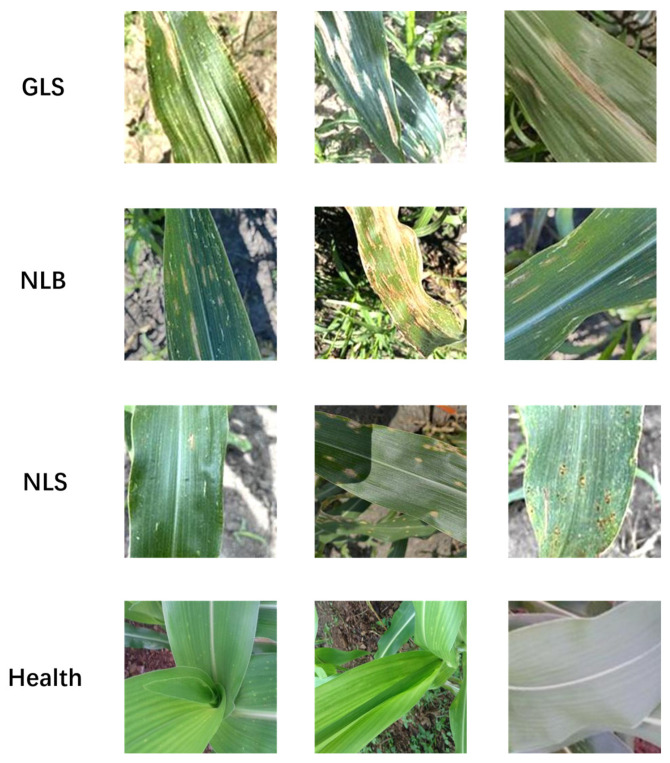
Maize leaves in a complex field background.

The target dataset of this paper’s experiment is maize leaf diseases. To address the issue of poor robustness in the trained model caused by image data with simple backgrounds, this paper opted to utilize maize leaf images captured under field conditions with complex backgrounds. Data sourced from (https://osf.io/s6ru5/ (accessed on 31 July 2023)). These images were captured through the 12-megapixel camera sensor on the iPhone 11 Pro smartphone. These images were originally sized at 3000 × 3000 pixels and maintained a 1:1 aspect ratio [25]. During the model training process, to ensure that the input image meets the model requirements, the resolution of the image was unified to 224 × 224. The dataset comprised healthy maize leaves along with images depicting three common maize leaf diseases in complex backgrounds, namely Northern Leaf Blight (NLB), Gray Leaf Spot (GLS), and Northern Leaf Spot (NLS) of maize (Figure 1), totaling 1902 maize leaf images. From Table 1, the original data are both limited in quantity and unevenly distributed. This may lead to a decrease in the model’s generalization ability, subsequently affecting the accuracy of evaluation results and hindering the model from being adequately trained. To address these issues, this study plans to employ data augmentation techniques to balance the quantity of samples across different categories, thereby enhancing the robustness of the model.

#### 2.1.2. Data Augmentation

**Figure 2:**
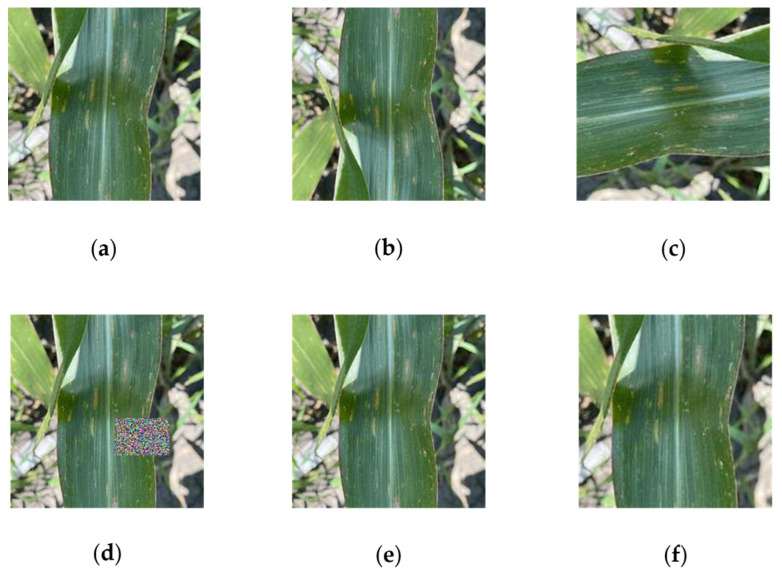
Data augmentation effect image. (**a**) Original image; (**b**) vertical flip: vertical flip probability set to 0.5; (**c**) random rotation: rotation probability set to 0.7, with a maximum angle of 10 degrees to the left and to the right; (**d**) region erasure: erasure probability set to 0.3, with the size of the erasure area being 0.3 of the image size; (**e**) brightness transformation: brightness adjustment probability set to 0.5, with the minimum value being 0.7 times and the maximum value being 1.3 times; (**f**) morphological amplification: magnification probability set to 0.3, with the minimum amplification factor being 1.1 times and the maximum being 1.6 times.

To ensure a relatively balanced number of samples across various categories in the dataset and enhance the performance and generalization ability of the model, a total of 4000 images were obtained through the random expansion and enhancement of the original 1902 images for training the research model. In this paper, the Augmentor tool in Python was employed for data augmentation, encompassing random rotation, vertical flipping, shape amplification, region erasure, and brightness transformation (Figure 2).

### 2.2. Construction of Identification Model of Maize Leaf Disease

#### 2.2.1. ShufflenetV2

**Figure 3:**
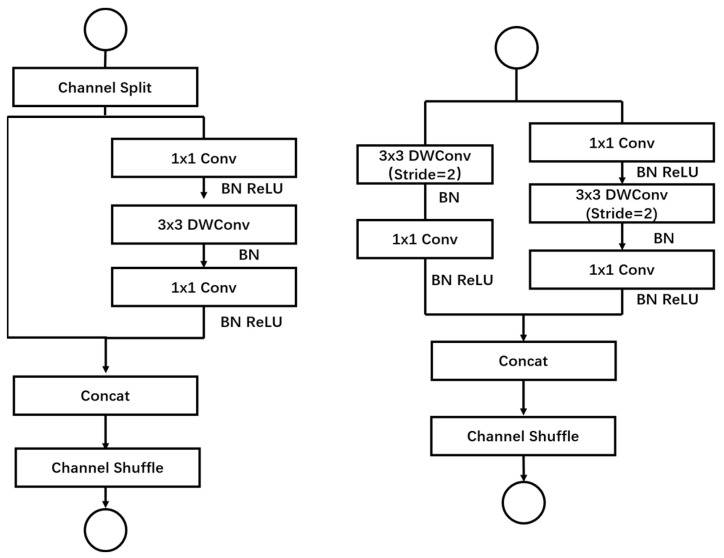
The basic unit of ShufflenetV2.

ShuffleNetV2, a lightweight convolutional neural network model proposed by MegVII [26], presents several advantages over traditional neural network models, including its lightweight, flexible, and highly efficient nature. The ShuffleNetV2 block introduces a Channel Split operation based on the ShuffleNetV1 block, dividing the input feature map into two blocks. In each block, half of the feature channels pass directly through the block and join the next block. This strategy reduces computations and parameters while increasing the number of feature channels, thereby enhancing network accuracy. Within the network module, some feature channels are passed directly to the next module without undergoing convolution calculations. In the subsampling layer of the network, instead of employing channel separation, each branch is created by copying the input, and subsampling operations with a step size of 2 are performed on each branch. Finally, the feature maps from all branches are concatenated, halving the spatial size of the feature maps while doubling the number of channels (Figure 3). This design effectively reduces computation while maintaining information richness and enhancing network expression capability [27]. Compared to the ShuffleNetV1 version, where ShuffleNetV2 adds a conv5 convolution before global pooling, ShuffleNetV2 enhances network performance and generalization. This model demonstrates strong feasibility, enabling rapid image processing and recognition on mobile devices while remaining lightweight. It holds promising prospects for applications in embedded computing, mobile terminals, and large-scale image recognition.

#### 2.2.2. Max Pooling

Max Pooling is a down-sampling operation utilized to decrease computing and storage demands. This layer is commonly employed to diminish the size of a feature map while preserving crucial feature information. By partitioning the input feature map into fixed-size rectangular regions and selecting the maximum value within each region as the output, maximum pooling effectively diminishes the spatial dimension of the feature map while retaining features with the strongest response [28]. In the formula, the maximum pool selects the maximum value within the locally acceptable domain F, as demonstrated in Equation (1).

(1) $$y=Max\left(x_{1},x_{2},\cdots ,x_{i}\right),\left(x_{i}\in F\right)$$

**Figure 4:**
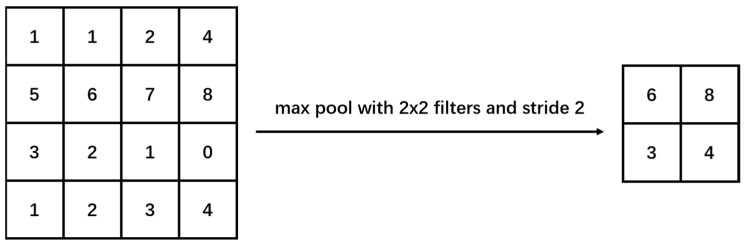
Illustration of Max Pooling.

The maximum value serves as a significant feature extracted by the convolutional layer. By retaining these crucial features and discarding unimportant ones, the interference of irrelevant information is minimized (Figure 4). The incorporation of a maximum pooling layer can augment the characterization capability of the model. Furthermore, the maximum pooling layer also aids in reducing computation by diminishing the size of the feature map, thereby rendering the model more lightweight.

In this paper, a Max Pooling layer is integrated into the ShuffleNetV2 network. This layer reduces the width and height of the feature map by half, gradually diminishing its size. It facilitates the extraction of key information from feature maps, the reduction in model overfitting, and the enhancement of the model’s generalization capability.

#### 2.2.3. SimAM Attention Module

**Figure 5:**
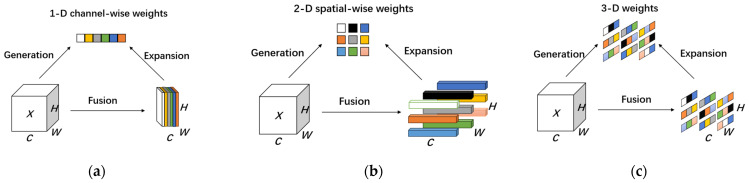
Comparisons of different attention steps. Most of the existing attention modules generate 1D or 2D weights from feature X and then expand the generated weights for channel (**a**) and spatial (**b**) attention. SimAM attention instead directly estimates 3D weights (**c**). In each subfigure, the same color denotes that a single scalar is employed for each channel, for the spatial location, or for each point on that feature.

Existing attention modules in computer vision typically focus on either the channel domain or spatial domain, corresponding to feature-based attention and spatial-based attention in the human brain, respectively. Traditional channel attention is one-dimensional, concentrating on the characteristics of different channels while treating all positions equally. Spatial attention, on the other hand, is two-dimensional, focusing on features at different locations while treating all channels equally. However, Yang et al. argue that computing three-dimensional values should be straightforward and allow modules to maintain lightweight properties [29]. Consequently, they proposed a simple yet effective attention mechanism called SimAM. SimAM can directly derive three values for the feature map without adding additional parameters, enabling the model to learn more discriminative neurons and improving the network’s feature extraction ability (Figure 5). Additionally, based on neuroscience theory, SimAM optimizes the energy function to mine the importance of neurons, enhancing the ability to extract important features while suppressing the interference of non-important features. Furthermore, SimAM’s operation is primarily based on the selection of an optimized energy function, avoiding excessive structural adjustments, and accelerating the calculation of attention weights. This allows the network to remain lightweight while better leveraging the effectiveness and flexibility of SimAM when integrated into our ShuffleNetV2 model.

In this paper, the SimAM attention mechanism is inserted into the basic network unit of ShuffleNet V2. This enhances the interaction between channels, enabling the model to better adapt to different input features, improve representation ability, and enhance classification performance. Additionally, the SimAM module assists the model in capturing important image features more efficiently and weighing these features to enhance recognition and classification accuracy. By introducing the SimAM module, ShuffleNetV2 can enhance its image processing and recognition capabilities while maintaining lightweight and efficient performance, thereby making the model more applicable to mobile devices and embedded systems.

#### 2.2.4. Improved ShufflenetV2

**Figure 6:**
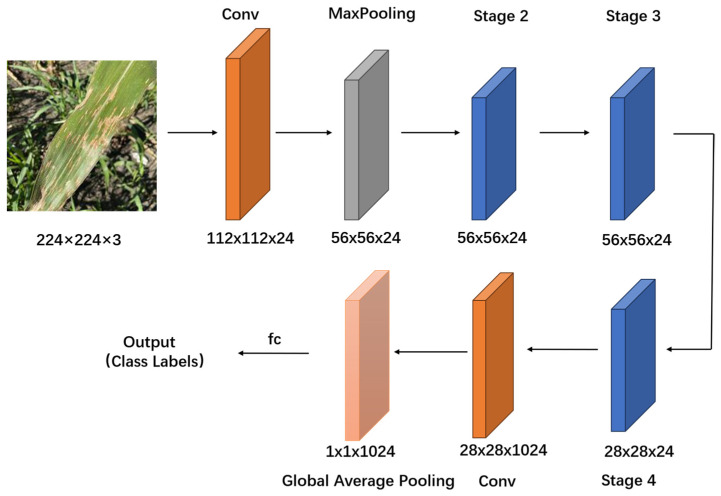
Structure of the SNMPF model. Input a 224 × 224 × 3 size image, first through a 3 × 3 convolution and then through the Max Pooling, followed by several Stage modules, and then through a 1 × 1 convolution. Finally, input through the full pooling layer (Global Pooling) and then connect to a fully connected layer to obtain the output.

Based on the characteristics of corn leaf disease images, ShuffleNetV2-0.5 was chosen as the baseline network and improved to create the SNMPF model. In the basic unit of the ShuffleNetV2 network, the stride in depth-wise (DW) convolution, which was originally set to 2 for down-sampling, is reduced to 1. Additionally, in the down-sampling module of the ShuffleNet model, the maximum pooling layer is employed instead of deep convolution for down-sampling. This enhancement aids in extracting crucial features from images, mitigating model overfitting, and enhancing the model’s generalization ability (Figure 6).

**Figure 7:**
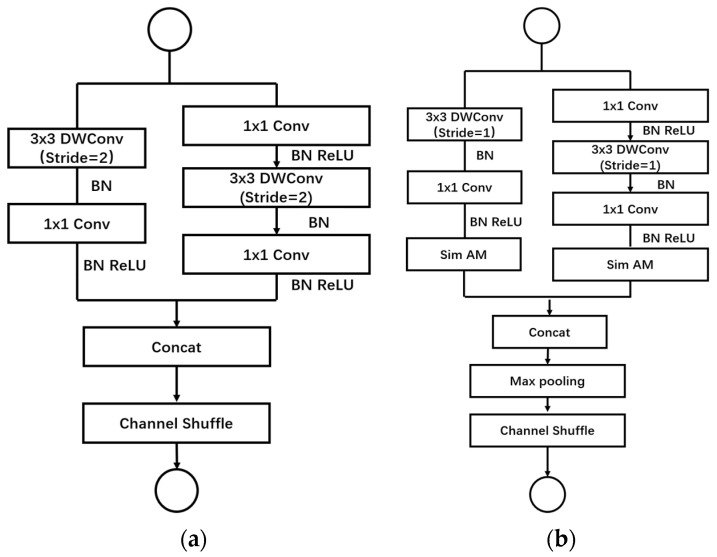
Comparison of the basic units of the initial network and the improved network. (**a**) Initial network; (**b**) improved network.

Given that the dataset used in this paper consists of corn leaf images captured under complex backgrounds, non-important features such as environmental backgrounds may interfere with model recognition. Therefore, SimAM attention was integrated into the ShuffleNetV2 network (Figure 7) to alleviate the interference of non-important features, such as complex environmental backgrounds, on model recognition.

### 2.3. Experimental Setup

The experiments were carried out on a 64-bit Windows 10 operating system using the Python programming language. The PyTorch framework was employed for network construction, training, and testing purposes. The computer utilized for these experiments is equipped with an Intel(R) Core (TM) i7-10510U CPU and has 8 GB of RAM. Additionally, it is complemented by an NVIDIA GeForce MX250 GPU for accelerated processing.

### 2.4. Training Hyperparameter Settings

In our experiments, we use the SGD optimizer to iteratively update model parameters to minimize the loss function. This choice was made due to the advantages of the SGD optimizer in terms of computational efficiency and memory requirements. Additionally, we set the following hyperparameters: epoch = 30, learning rate = 0.01, and batch size = 4. These hyperparameters were carefully selected to maximize the effectiveness and accuracy of our model. By choosing these hyperparameters, we aimed to optimize our deep learning model and enhance its performance in recognition tasks.

### 2.5. Model Evaluation

In this paper, our main evaluation metrics include accuracy, model loss, and the size of the model. Accuracy (A) is calculated using Equation (2), while model loss is computed according to Equation (3). Accuracy is defined as the ratio of correctly recognized samples to the total number of samples.

(2) $$A=\frac{TP+TN}{TP+TN+FP+FN}\times 100\%$$

TP (True Positive): The number of positive samples correctly predicted as positive by the model. In other words, the model successfully detected a positive sample.

FP (False Positive): The number of negative samples incorrectly predicted as positive by the model. In other words, the model incorrectly predicted negative samples as positive samples.

TN (True Negative): The number of negative samples correctly predicted as negative by the model. In other words, the model correctly determined negative samples.

FN (False Negative): The number of positive samples incorrectly predicted as negative by the model. In other words, the model incorrectly predicted positive samples as negative samples.

The loss function provided in Equation (3) is a formulation commonly used in margin-based methods, such as contrastive loss or triplet loss, for tasks like metric learning or Siamese network training.

(3) $$Loss=yd^{2}+(1-y)\cdot {max(0,\;margin-d)}^{2}$$

In this equation, the following variables are defined:

d: Represents the Euclidean distance between two samples.

y: Represents the label indicating whether the two samples match. y = 1 indicates that the two samples belong to the same category, while y = 0 indicates that the two samples belong to different categories.

margin: Denotes the threshold.

The loss function encourages the model to learn embeddings such that the distance between samples from the same category is minimized while ensuring that the distance between samples from different categories is larger than the margin. This facilitates a better discrimination between classes in the learned feature space.

## 3. Results

### 3.1. Results on Data Enhanced Analytics

**Figure 8:**
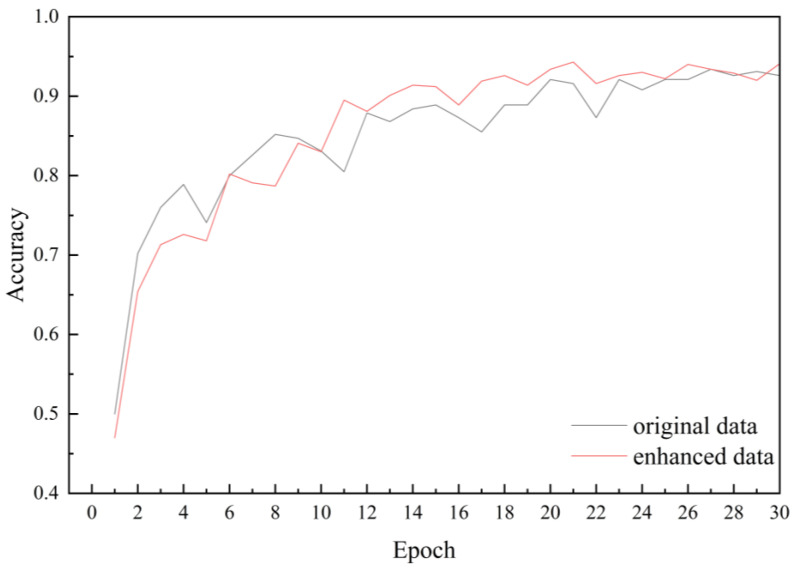
Accuracy curves before and after data augmentation.

ShuffleNetV2 was utilized to train both the original maize leaf disease dataset and its augmented counterpart to assess the viability of data augmentation. It was observed that the performance of the ShuffleNetV2 model on the two datasets exhibited the following trend: the augmented data outperformed the raw data. When using an enhanced dataset, the accuracy of the model increased from 93.40% to 94.30%, an increase of 1.1 percentage points, while the loss slightly decreased from 0.416 to 0.414. This means that data augmentation effectively improves the performance of the model (Table 2). As the number of epochs increased, the recognition accuracy of the model trained on augmented data demonstrated improvement (Figure 8).

**Figure 9:**
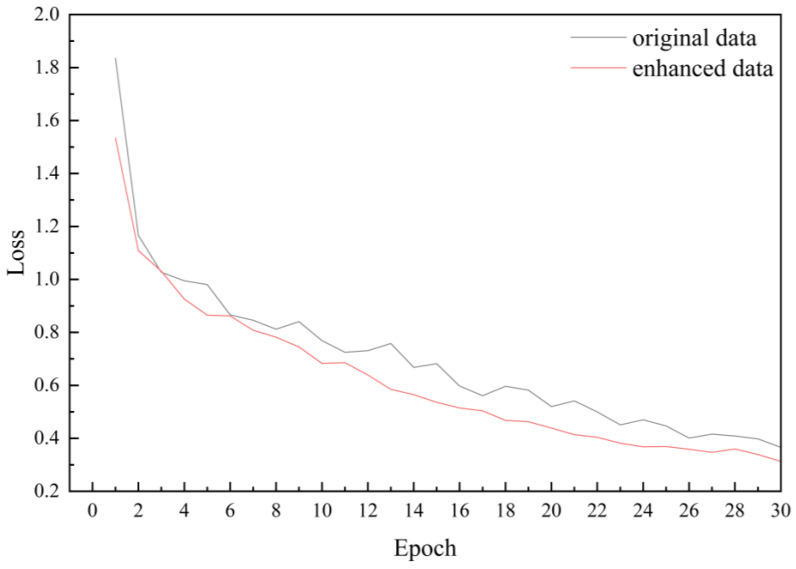
Loss curves before and after data augmentation.

It is worth noting that after data augmentation, the overall loss decreased compared to the original data, and the convergence speed accelerated (Figure 9). Furthermore, as epochs progressed, the enhanced data notably enhanced model accuracy, affirming the viability of data augmentation. Remarkable outcomes were achieved in enhancing model performance through data augmentation, as evidenced by the gradual reduction in the loss function and the progressive increase in accuracy. These findings underscore the positive impact of data augmentation on model training and performance.

### 3.2. Results of the Ablation Test

**Figure 10:**
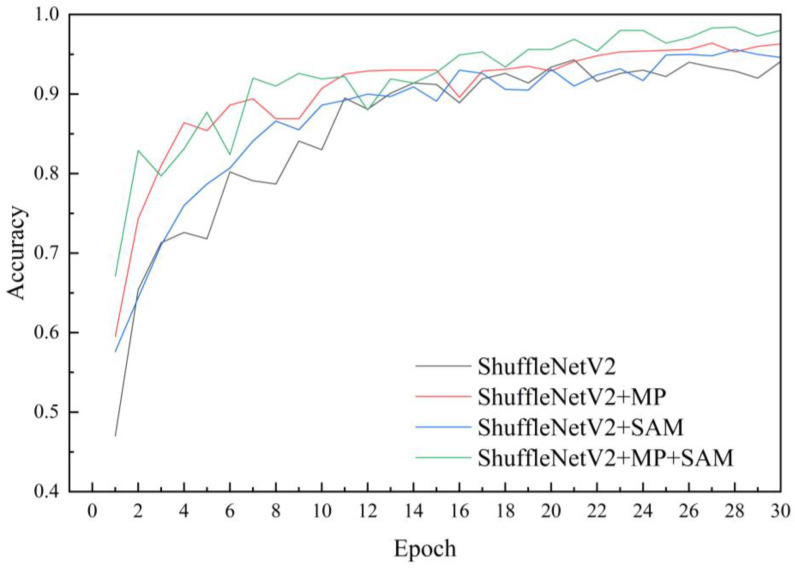
Accuracy curve of ablation test. This figure illustrates how adding different modules (MP, SAM, MP + SAM) affects the recognition accuracy of the model. The *x*-axis represents epoch, and the *y*-axis represents accuracy. The curve indicates that the model with the addition of MP + SAM has the best recognition performance.

**Figure 11:**
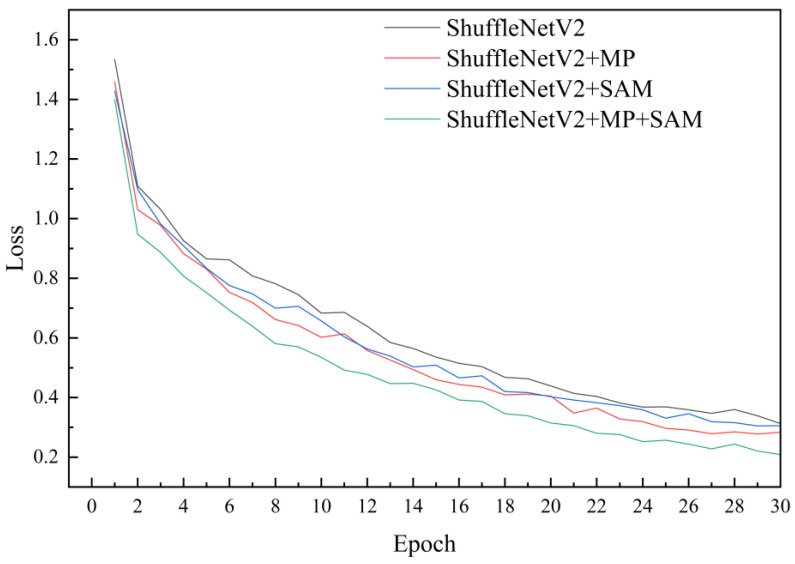
Loss curve of ablation test. This figure illustrates how adding different modules (MP, SAM, MP + SAM) affects the generalization ability of the model. The *x*-axis represents epoch, and the *y*-axis represents loss. The curve indicates that the model with the addition of MP + SAM fits the training data best.

To validate the impact of various enhancements proposed in this paper on model performance, an ablation test was conducted for analysis. Throughout the experiments, consistency in test conditions was maintained, with only one enhancement altered in each experiment to assess its influence on model performance. Four models were evaluated: ShuffleNetV2, ShuffleNetV2 + Max Pooling (MP), ShuffleNetV2 + SimAM (SAM), and ShuffleNetV2 + Max Pooling (MP) + SimAM (SAM). Results revealed that incorporating Max Pooling into the ShuffleNetV2 network without increasing model complexity led to a 2.1 percentage point improvement in recognition accuracy for corn leaf diseases in complex environments, accompanied by a reduction in model loss by 0.135. The introduction of the SimAM module increased model size by only 0.06 MB, enhancing accuracy by 1.3 percentage points (Table 3). These findings indicate that the addition of SimAM resulted in improved accuracy and reduced loss, underscoring the effectiveness of model enhancements (Figure 10). The model incorporating both Max Pooling and SimAM achieved the highest performance (Figure 11). The synergistic effect of these enhancements did not adversely affect the model but rather boosted its accuracy. The enhanced model achieved an accuracy of 98.40%, surpassing the original model by 4.1 percentage points, with a loss of 0.228, 0.186 lower than that of the original model. Although the size of the improved model slightly increased, it remained lightweight.

### 3.3. Results of the Attention Test

To further validate the feasibility of incorporating SimAM attention in this paper, comparative experiments were conducted by adding SimAM, SE, ECA, EMA, and CSAM attention mechanisms to the ShuffleNetV2 model. These experiments aimed to analyze the effects of different attention mechanisms on the recognition and detection of maize leaf diseases amidst complex backgrounds. The results revealed that following the integration of SEM, ECA, EMA, CSAM, and SimAM attention mechanisms, the accuracy of maize leaf disease recognition by the ShuffleNetV2 model improved by 0.3%, 2.1%, 2.6%, 3.5%, and 4.1% (Table 4), respectively. Notably, the introduction of an attention mechanism effectively enhanced the model’s capability to focus on crucial lesion features within complex backgrounds. Particularly, the introduction of the SimAM model exhibited the most significant enhancement in recognition performance.

Conversely, the introduction of the SE attention mechanism resulted in a decrease in model loss by 1.9 percentage points. Moreover, the integration of attention mechanisms led to an increase in the size of the model compared to the original ShuffleNetV2 model. Specifically, the ECA attention mechanism exhibited the smallest model size increase of 1.509 MB, followed by the SimAM attention mechanism with 1.556 MB, while the CSAM attention mechanism demonstrated the largest model size increase of 2.017 MB (Table 4). Consequently, SimAM attention emerged as a pivotal factor in enhancing the model’s lightweight performance while achieving the highest accuracy gain, highlighting the significance of integrating attention mechanisms to enhance the performance of lightweight models in complex recognition tasks such as maize leaf disease identification.

**Figure 12:**
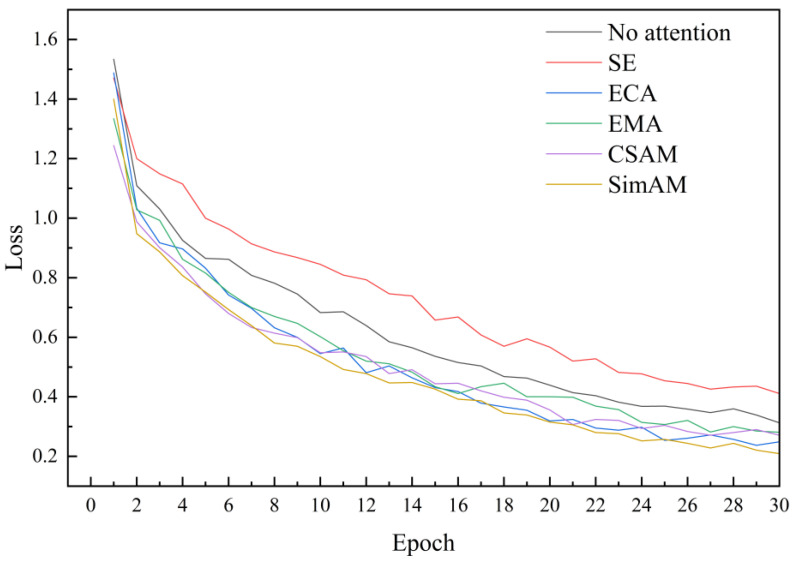
Attention test model loss curve. This figure depicts the loss of the model when adding different attention mechanisms (SE, ECA, EMA, CSAM, SimAM). The *x*-axis represents epoch, and the *y*-axis represents loss. The curve indicates that the model with added SimAM attention mechanism has the smallest loss.

Subsequently, we draw the loss curve of the model to analyze the impact of different attention mechanisms on the robustness of the model. It was observed that the loss values of the models gradually decreased and stabilized with an increase in the number of model training iterations. By introducing ECA, EMA, CSAM, and SimAM attention mechanisms, the loss of the model was reduced. Especially after introducing the SimAM attention mechanism, the model exhibited the lowest loss value and significantly improved robustness. However, SE attention led to an increase in model loss (Figure 12).

### 3.4. Results of Model Comparison Test

To explore the advantages of the lightweight model developed in this paper for recognizing maize leaf diseases in complex scenarios, comparisons were drawn with contemporary network modeling algorithms. In the task of maize leaf disease recognition and detection, SNMPF achieved the highest recognition accuracy of 98.4%. DenseNet and EfficientNet performed similarly, while the MobileViT model exhibited the lowest recognition accuracy at only 64.7%. Furthermore, the model loss of MobileViT was notably high at 0.839 compared to several other models (Table 5). Although EfficientNetV2 is a widely optimized and efficient model, its performance in the recognition task of this paper was not as expected and even worse than EfficientNet. The main reason may be that EfficientNetV2 was originally designed to perform well on large and diverse datasets. However, our dataset size is relatively small, which may result in EfficientNetV2 not being able to fully leverage its advantages. In contrast, EfficientNet had better adaptability on the dataset in this paper.

**Figure 13:**
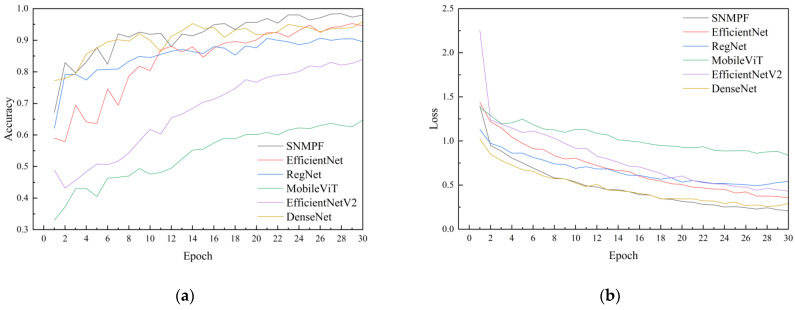
(**a**) Accuracy curves of different models; (**b**) loss curves of different models.

The SNMPF model, built upon the enhanced ShuffleNetV2 architecture proposed in this paper, demonstrated superior performance. It achieved the highest recognition accuracy for maize leaf disease under complex backgrounds with minimal model loss (Figure 13). Moreover, the model size of SNMPF was a mere 1.56 MB, which was less than half of MobileViT’s model size and even less than one-tenth of other models.

### 3.5. Visual Presentation of Model Prediction Results

The SNMPF model was used to predict the test image and then the activation heat map (Grad-CAM) technique was used to visualize the prediction of the model. Grad-CAM can display the recognition focus area of the model by evaluating the contribution of each region in the image to the prediction results. In this way, we can more accurately understand the features that the model focuses on, which can improve the understanding of the model’s prediction results and the interpretability of the model.

**Figure 14:**
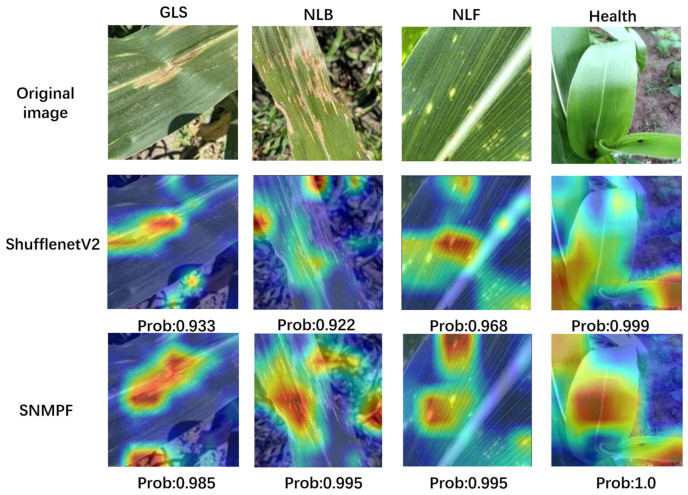
Visualization results of SNMPF in identifying maize leaf diseases. Different species of diseases have different thermogram characteristics, and the closer the color is to red, the more it plays a role in decision-making for the identification of that species.

The results revealed that for the four types of maize leaf images—GLS, NLB, NLF, and Health—the original ShuffleNetV2 model correctly predicted the disease with probabilities of 0.933, 0.922, 0.968, and 0.999, respectively. The SNMPF model developed in this paper achieved correct predictions with probabilities of 0.985, 0.995, 0.995, and 1.0, respectively (Figure 14). Even in the presence of complex background interference, SNMPF could still effectively capture the positions of maize leaf lesions and extract their features. It demonstrated relatively high recognition accuracy for the same type of leaf disease, enabling better focus on the identified lesion areas and thus reducing the impact of complex backgrounds.

This result further proves the effectiveness of the model improvement measures proposed in this paper, as well as the excellent performance of the SNMPF model in corn leaf disease recognition tasks in complex backgrounds, providing useful references for corn leaf disease recognition on mobile devices.

## 4. Discussion

After introducing the Maximum Pooling layer into ShuffleNetV2, the accuracy of the model in identifying corn leaf disease improved by 2.1 percentage points, and the model’s loss was also reduced. This is because the max pooling layer can select the maximum value in each region, thereby reducing the size of the feature map while retaining the most significant feature information, thereby improving the model’s generalization ability [30]. Introducing attention mechanisms enables the model to focus more sensitively on disease areas, thereby aiding in distinguishing various leaf disease images [31]. After introducing the SimAM attention mechanism in ShuffleNetV2, the model performance has been improved. SimAM can help models focus more on important features [32], thereby improving their ability to extract key information from images. Meanwhile, when both the max pooling layer and SimAM attention mechanism are added to the model, the performance of the model is further improved. This is because the max pooling layer effectively reduces the dimensionality of the feature map and preserves the most important information, while the SimAM attention mechanism further enhances the model’s perception of key features, making it more accurate in identifying and classifying objects in the image. Overall, by combining these two mechanisms, the model’s ability to extract and utilize features has been further enhanced, thereby improving overall performance.

To assess the feasibility of SimAM attention, a comparison was conducted with SE, ECA, EMA, and CSAM attention mechanisms within the ShuffleNetV2 model [33,34,35,36]. The results showed that after introducing attention mechanisms such as SE, ECA, EMA, CSAM, and SimAM, the recognition accuracy of the model was improved to varying degrees. These attention mechanisms help the model focus on the lesion area, thereby significantly improving the performance of the model [37]. However, after introducing the SE attention mechanism, the model’s loss increases. This may be because SE introduces additional parameters, making the model more complex and leading to overfitting [38].

Furthermore, the recognition effects of SNMPF, EfficientNet, MobileViT, EfficientNetV2, RegNet, and DenseNet models on maize leaf diseases were compared [39,40,41,42,43]. Experimental results showed that EfficientNetV2 had the largest model size. However, large networks have high hardware requirements on computers and mobile devices, which is not conducive to a wide range of applications. Moreover, EfficientNetV2 had the worst recognition effect on maize leaf diseases, and the model loss value was also relatively large. This indicates that there is an overfitting problem [44], and EfficientNetV2 needs to be further optimized and adjusted for the identification and application of corn leaf diseases. The SNMPF model achieved the best recognition effect, and the highest recognition accuracy reaches 98.4%. Compared with the other models, the SNMPF model had the highest recognition accuracy and the smallest loss. The recognition accuracy is improved by 4.1%. This improvement was attributed to the addition of max pooling, which reduced model oversensitivity to feature locations, enhanced robustness [38], and minimized interference from complex backgrounds. Coupled with the SimAM attention mechanism, recognition accuracy was further improved.

Finally, comparing the prediction results of SNMPF and ShuffleNetV2 models for different types of maize leaf diseases, it can be observed that ShuffleNetV2 identifies some non-diseased areas as diseased areas, while the SNMPF model focuses more on diseased areas, reduces environmental impact, and improves the probability of accurate prediction [45]. These results further validate the effectiveness of the improvement measures in this article.

This paper verified the feasibility of ShuffleNetV2 in maize leaf disease recognition, providing technical support for deploying maize leaf disease recognition models on mobile devices. Due to limited public data on corn leaf disease, there are still limitations in this study, namely, the dataset used in this study is relatively limited. By comparing the model training results of the pre- and post-enhanced datasets, a large amount of data is beneficial for optimizing model performance. In the future, with the application of various image recognition technologies in the field, more types and quantities of corn leaf disease data images will be collected [46], which can increase the types of corn leaf disease recognition, further improve the recognition accuracy and performance of the model, and enhance the robustness and generalization ability of the model [47].

## 5. Conclusions

In this paper, we addressed the issues of low efficiency in traditional manual identification methods and the challenge of deploying existing recognition models on mobile devices due to their large size. We proposed a maize leaf disease recognition solution based on the improved lightweight convolutional neural network ShuffleNetV2. Focusing on maize leaf images in complex backgrounds, we improved the ShuffleNetV2 model by introducing max pooling layers to replace deep convolutional layers for down sampling, adding attention mechanisms, optimizing network structures, and we presented the SNMPF model.

The results indicate that the addition of max pooling layers is effective in improving the model’s ability to recognize maize leaf diseases. Introducing attention mechanisms further enhances ShuffleNetV2’s discriminative power in feature extraction and the classification of maize leaf diseases. After incorporating attention modules such as SE, ECA, EMA, CSAM, and SimAM, the accuracy of the model’s recognition is enhanced, with the model achieving optimal recognition performance after introducing SimAM. In the task of maize leaf disease recognition under complex backgrounds, the SNMPF model proposed in this paper outperforms other traditional neural network models, achieving a recognition accuracy of 98.4%. Additionally, its model size is only 1.56 MB, making it suitable for deployment on small mobile devices.

In summary, this paper provides new technical means for the recognition and detection of maize leaf diseases, with potential applications in field environments in the future. Since this paper only focuses on recognizing three common types of maize leaf diseases, further improvements are needed in future research. Future studies will enrich image data to recognize more types of leaf diseases and deploy the SNMPF model on mobile devices to achieve the real-time recognition and detection of maize leaf diseases. Therefore, enabling the timely detection of maize leaf diseases can foster the development of precision agriculture.

## Figures and Tables

**Table 1:** Data distribution.

Image Type	Number of Original Image	Number of Enhanced Image	Label
Northern Leaf Blight	497	1000	NLB
Healthy Leaves	331	1000	Health
Gray Leaf Spot	523	1000	GLS
Northern Leaf Spot	551	1000	NLS

**Table 2:** Comparison before and after data augmentation.

Data Set	Accuracy	Loss	Model Size/MB
Original date	93.40%	0.416	1.50
Enhanced date	94.30%	0.414	1.50

**Table 3:** Comparison of ablation tests.

Model	Accuracy	Loss	Model Size/MB
ShuffleNetV2	94.30%	0.414	1.50
ShuffleNetV2 + MP	96.40%	0.279	1.50
ShuffleNetV2 + SAM	95.60%	0.316	1.56
ShuffleNetV2 + MP + SAM	98.40%	0.228	1.56

**Table 4:** Comparison of different attentions.

Attention	Accuracy	Loss	Model Size/MB
No attention	94.30%	0.414	1.499
SE	94.60%	0.433	1.644
ECA	96.40%	0.298	1.509
EMA	96.90%	0.285	1.587
CSAM	97.80%	0.271	2.017
SimAM	98.40%	0.244	1.556

**Table 5:** Comparison of different models.

Model	Accuracy	Loss	Model Size/MB
SNMPF	98.4%	0.228	1.56
EfficientNet	95.3%	0.369	15.98
RegNet	90.7%	0.504	15.49
MobileViT	64.7%	0.839	3.86
EfficientNetV2	83.9%	0.431	79.73
DenseNet	95.9%	0.290	27.78

## Data Availability

Data supporting the findings of this paper are available from the corresponding authors.
